# Spirituality and psychological capital as predictors of psychological well-being in adults in Ecuador and Peru

**DOI:** 10.3389/fpsyg.2025.1620382

**Published:** 2025-08-20

**Authors:** Víctor Manuel López-Guerra, Dolores Lucia Quinde, Sandra Guevara-Mora, Karina Ocampo-Vásquez, Wilson Guillermo Siguenza-Campoverde, Cristina Díaz de la Cruz, Segundo Francisco Vivanco-Rios, Susan Cristy Rodríguez-Balcázar, José Melanio Ramírez-Alva

**Affiliations:** ^1^Department of Psychology, Universidad Técnica Particular De Loja, Loja, Ecuador; ^2^General Directorate of University Missions, Universidad Técnica Particular De Loja, Loja, Ecuador; ^3^Psychology Study Program, Universidad Privada Antenor Orrego, Trujillo, Peru; ^4^Psychology Study Program, Universidad Privada Antenor Orrego, Piura, Peru

**Keywords:** psychological well-being, spirituality, psychological capital, Ecuador, Peru

## Abstract

**Background:**

Several studies have indicated a positive association between positive psychological resources–such as spirituality and psychological capital–and psychological well-being. However, the specific nature of these relationships remains poorly understood.

**Objective:**

The purpose of this study was to examine, through a model of structural equations, the influence of spirituality and psychological capital on psychological well-being, as well as to explore the relationship between these two predictor variables.

**Method:**

The sample consisted of 1,044 adults living in Ecuador and Peru, aged between 18 and 71 years (*M* = 24; SD = 7.77), of whom 64.8% were women. The Ryff Psychological Well-being Scale (Ryff, 1989), the Parsian and Dunning Spirituality Scale (Parsian and Dunning, 2009), and a psychological capital questionnaire were used.

**Results:**

Structural analyses showed a good fit of the proposed model according to the main goodness-of-fit indices. Psychological capital emerged as the most robust predictor of psychological well-being, explaining 13% of its variance, followed by spirituality. Likewise, a positive association was also evident between spirituality and psychological capital. Overall, the findings suggest that higher levels of these positive resources are related to greater psychological well-being.

**Conclusion:**

The results provide relevant empirical evidence on the promoting role of positive internal resources in psychological well-being, especially in Latin American contexts. The theoretical and practical implications are discussed, the study’s limitations are acknowledged, and future research is proposed.

## 1 Introduction

Positive psychology investigates human functioning from an optimistic perspective, encompassing concepts related to happiness, well-being, and positive approaches to mental health. Among these is psychological well-being (PWB), a construct studied from two perspectives (Jiménez-Lira et al., 2020). On the one hand, there is the hedonistic approach that emphasizes subjective happiness, that is, satisfaction with life, a positive mood, and an absence of negative mood (Nogueira et al., 2023; Ryff et al., 2021; Rondón et al., 2024); on the other hand, the eudaimonic approach emphasizes that well-being is not limited to a moment of pleasure but rather implies personal growth and the commitment that the individual assumes in the face of life’s challenges (Arce et al., 2023; Ryff, 2023; Rondón et al., 2024).

From a eudaimonic perspective, the World Health Organization (World Health Organization, 2022) recognizes psychological well-being (PWB) as a fundamental component of comprehensive health. It is understood as a state in which the individual is aware of their own capabilities, can effectively cope with daily stresses, perform productively, and actively contribute to the development of their community (Bożek et al., 2020). In line with this approach, the multidimensional model proposed by Ryff (1989, 2018) has established itself as one of the most influential theoretical frameworks for the assessment of PWB. This model proposes six interrelated dimensions that reflect optimal psychological functioning: self-acceptance, positive relationships with others, autonomy, management of the environment, personal growth, and purpose in life.

Self-acceptance refers to the positive assessment one has of oneself and the awareness of one’s own actions, feelings, motives, and opportunities for improvement (Ryff, 1989; Ryff, 2018). Positive relationships include close, trusting bonds where concern for the well-being of others is expressed (Ryff, 1989; Ryff and Keyes, 1995; Ryff, 2014). Personal growth is oriented toward continuous improvement and the development of the potential of individuals (Ryff, 1989; Ryff, 2018). Autonomy is related to independence, self-determination, and the ability to self-evaluate based on personal standards (Ryff, 2014; Ryff, 2018). Environmental management refers to the ability to effectively grapple with the environment and control various external activities, taking advantage of the opportunities offered by the context (Ryff, 1989; Ryff, 2018). Finally, the purpose in life guides the establishment of goals and definition of objectives that individuals seek to achieve (Ryff, 1989; Ryff, 2018).

The PWB has been studied in diverse contexts and populations, showing significant relationships with multiple variables, such as academic performance (Barrera Hernández et al., 2019), mental health (Bożek et al., 2020), coping with work stress (Malekitabar et al., 2016), and resilience in the face of adversity (García Orbe et al., 2024). Along these lines, two constructs emerge as key resources in promoting well-being: spirituality and psychological capital (PsyCap).

Spirituality has been identified as a key variable in promoting psychological well-being. In recent years, this field has sparked growing interest in scientific research, consolidating its position as a relevant and expanding area. A bibliometric analysis of the Web of Science database (2014–2025) revealed a significant increase in publications on “spirituality,” reaching more than 19,962 studies in the last decade and practically doubling the annual number of publications since 2014.

According to Joseph et al. (2017), spirituality can be understood as a natural construct through which human beings seek to connect both with a higher being and with themselves. In this sense, Koenig et al. (2023) define it as the set of beliefs, practices, and individual ways of being destined to facilitate the union or experience of the divine or transcendent. Likewise, it is understood as the search for meaning in life, self-realization, and connection with the inner self and the universe (Parsian and Dunning, 2009). This conceptualization of spirituality implies a connection with beliefs, values, and practices, both personal and religious, that give meaning to life and inspire and motivate people to reach their full potential (Tanyi, 2002). This connection translates into faith, hope, peace, and empowerment, resulting in joy, forgiveness, conscience, acceptance of difficulties and mortality, greater physical and emotional well-being, and the ability to transcend the limitations of existence. It can also be understood as a dynamic process in constant exploration of new experiences that involves the recognition of the ultimate limits of existence and the search for a deeper purpose in life (Bożek et al., 2020). According to Parsian and Dunning (2009), spirituality is composed of four dimensions: Self-awareness, focused on people’s knowledge of themselves in terms of thoughts, feelings, and behaviors; spiritual beliefs, which reflect the value assigned to the quality of spiritual beliefs and their influence on decision-making; spiritual practices that explore experiences that strengthen spiritual connection; and spiritual needs that encompass the search for purpose and meaning in life, the search for inner peace, and the desire to connect with something greater than oneself.

Recent studies have shown the value of spirituality as a coping strategy and its contribution to a better quality of life, happiness, fewer depressive symptoms, greater resilience and psychological well-being (Caccia and Elgier, 2020; Bożek et al., 2020; Aprilia et al., 2024; Castañón-Valdivia et al., 2024).

On the other hand, psychological capital is defined as a state of positive psychological development characterized by self-efficacy, optimism, hope, and resilience (Luthans et al., 2004; King et al., 2020). Self-efficacy, which refers to self-confidence, consists of the individual’s ability to mobilize their cognitive and motivational resources, skills, and actions to achieve success in challenging tasks within a given context (Luthans et al., 2004; Luthans et al., 2015). Optimism involves interpreting present and future events in a positive way (Seligman, 2002; Luthans et al., 2015). Regarding hope, it is considered a positive motivational state that includes persistence toward goals and the ability to adjust them if necessary to achieve success (Luthans et al., 2015). As for resilience, it is the ability of individuals to recover from adverse situations to achieve success (Luthans et al., 2015; King et al., 2020). Resilient people accept reality, find meaning in life, and adapt to changes (Coutu, 2002).

Unlike personality traits, these components of psychological capital are considered dynamic psychological states, susceptible to strengthening through psychoeducational interventions and human development programs (Luthans et al., 2015; King et al., 2020). Various studies have demonstrated their positive impact on job satisfaction (Kurt and Demirbolat, 2019; Ho and Chan, 2022), stress coping, mental health and subjective well-being (Kurt and Demirbolat, 2019; Ho and Chan, 2022; Jones et al., 2023; Saman and Wirawan, 2024).

Recent empirical research has demonstrated a positive association between spirituality and various components of psychological capital (PsyCap), including hope, self-efficacy, resilience, and optimism. Spirituality involves not only a connection with the transcendent but also an active search for meaning, life purpose, and self-awareness (Koenig et al., 2023; Parsian and Dunning, 2009). Such experiences of introspection and transcendence contribute to the development of positive beliefs about one’s own abilities (self-efficacy), a hopeful outlook on the future (hope), a positive interpretation of challenges (optimism), and the capacity to cope with adversity (resilience) (Joseph et al., 2017; Parviniannasab et al., 2022).

Empirical studies have shown that specific dimensions of spirituality, such as spiritual self-awareness and existential needs, are significantly related to PsyCap components (Caccia and Elgier, 2020; Shamsi Lahijani et al., 2024). Moreover, recent findings suggest that spirituality may serve as a facilitator or catalyst for the development of positive psychological resources, providing interpretative frameworks that promote resilience and emotional regulation (Bożek et al., 2020; Pawar, 2016).

For instance, Caccia and Elgier (2020) found that among Argentine adolescents, spirituality was significantly associated with resilience (*r* = 0.61) and life satisfaction (*r* = 0.56), suggesting a protective role during this developmental stage. Similarly, Parviniannasab et al. (2022) reported that, in a sample of Iranian nursing students, spiritual well-being significantly predicted psychological capital (β = 0.28) and that PsyCap partially mediated its effect on mental health, indicating an indirect pathway of influence. Furthermore, Shamsi Lahijani et al. (2024) observed that spiritual health was directly related to both resilience (β = 0.34) and self-efficacy (β = 0.85), with self-efficacy also being influenced by resilience (β = 0.87). Collectively, these findings support the role of spirituality as a potential driver for the development of positive psychological resources, particularly among young people and individuals undergoing vocational training.

Despite theoretical and empirical advances, significant gaps in the literature remain. Most studies address spirituality and psychological capital as isolated predictors of psychological well-being, without integrating both constructs into joint explanatory models. Furthermore, there is a paucity of empirical research in Latin American contexts, particularly in countries like Ecuador and Peru, where the role of these positive psychological resources may be influenced by cultural, social, and economic factors specific to the region.

From an applied perspective, understanding the combined impact of psychological capital and spirituality on psychological well-being can guide the design of comprehensive psychoeducational interventions aimed at strengthening human development, preventing emotional distress, and fostering meaningful lives in diverse populations. With this in mind, this study aims to analyze the predictive role of psychological capital and spirituality on psychological well-being in a sample of adults from Ecuador and Peru.

The hypotheses raised are the following:

H1: Spirituality has a direct and positive effect on psychological well-being.

H2: Psychological capital has a direct and positive effect on psychological well-being.

H3: Spirituality has a direct and positive effect on psychological capital.

## 2 Materials and methods

### 2.1 Type of study and research design

The type of research was non-experimental and explanatory, with a cross-sectional design, based on a structural equation model (Kerlinger and Lee, 2002).

### 2.2 Participants

A total of 1,700 adults residing in Ecuador and Peru initially participated in the study through an online survey. The inclusion criteria were: being at least 18 years old, residing in Ecuador or Peru, having internet access, possessing the cognitive ability to autonomously complete the questionnaires, and providing informed consent to participate voluntarily. Participants with incomplete responses were excluded, resulting in a final sample of 1,044 individuals.

Although a non-probabilistic convenience sampling strategy was used, several actions were taken to reduce potential selection bias. The assessment battery was distributed online through multiple channels, including webinars organized by the Wellbeing Unit of the Universidad Técnica Particular Cristina Diaz de la Cruz Loja as part of the World Mental Health Day activities. Additionally, the general population was invited to participate through mass media campaigns, including television and radio announcements in both Ecuador and Peru. These dissemination efforts enabled the inclusion of not only university students but also working adults and individuals outside the educational system. Nevertheless, the final sample was predominantly composed of Ecuadorian university students, reflecting the greater reach and engagement achieved within this subgroup.

The participants’ average age was 24 years (SD = 7.77), with ages ranging from 18 to 71 years. In terms of sociodemographic characteristics, 64.8% identified as women, 85.0% were single, 77.7% were university students, and 82.8% reported not having children. Moreover, 56.4% were unemployed, and 71.0% perceived themselves as belonging to the middle socioeconomic class. Regarding nationality, 67.8% of participants were Ecuadorian, while 32.2% were Peruvian.

### 2.3 Instruments

#### 2.3.1 Sociodemographic questionnaire

An *ad hoc* questionnaire was designed to collect relevant information on the participants’ personal and contextual characteristics. It included questions on age, sex, marital status, nationality, country of residence, academic status (whether currently enrolled in university), self-perceived socioeconomic status, employment status, and the presence or absence of children.

#### 2.3.2 Ryff psychological well-being scale

The Spanish version of the original scale proposed by Ryff (1989), adapted by Díaz et al. (2006), was used. It is composed of 39 items distributed in six dimensions: self-acceptance (items 1, 7, 13, 19, 25 and 31; α = 0.84), positive relationships (items 2, 8, 14, 20, 26 and 32; α = 0.78), purpose in life (items 6, 12, 17, 18, 23 and 29; α = 0.70), personal growth (items 24, 30, 34, 35, 36, 37 and 38; α = 0.71), autonomy (items 3, 4, 9, 10, 15, 21, 27 and 33; α = 0.70) and environmental management (items 5, 11, 16, 22, 28 and 39; α = 0.82). The total scale presented adequate internal consistency (α = 0.86).

Items are answered on a six-point Likert scale ranging from “strongly disagree” to “strongly agree,” with higher scores reflecting higher levels of psychological well-being. Regarding its construct validity, the confirmatory factor analysis conducted by Díaz et al. (2006) supported the theoretically proposed six-factor structure. In that study, additional reliability indices were reported in both Cronbach’s alpha and McDonald’s omega for each dimension: self-acceptance (α = 0.75; ω = 0.74), positive relationships (α = 0.69; ω = 0.70), purpose in life (α = 0.81; ω = 0.82), personal growth (α = 0.68; ω = 0.69), autonomy (α = 0.69; ω = 0.72), and environmental management (α = 0.65; ω = 0.65).

#### 2.3.3 Spirituality scale

The scale developed by Parsian and Dunning (2009) was used, translated into Spanish and culturally adapted by Díaz Heredia et al. (2012). This instrument consists of 29 items distributed in four dimensions: self-awareness (items 1–10; α = 0.84), importance of spiritual beliefs in life (items 11–14; α = 0.91), spiritual practices (items 15–20; α = 0.76), and spiritual needs (items 21–29; α = 0.79). Responses were recorded on a four-point Likert scale, ranging from “strongly disagree” (1) to “strongly agree” (4). The internal consistency of the total scale was excellent (α = 0.88). Previous studies support its factorial validity through exploratory and confirmatory analyses that showed a four-factor structure, explaining 52.6% of the total variance. In the present study, optimal reliability indices were obtained: total scale (α = 0.94; ω = 0.94), self-awareness (α = 0.89; ω = 0.89), spiritual beliefs (α = 0.91; ω = 0.91), spiritual practices (α = 0.82; ω = 0.82) and spiritual needs (α = 0.85; ω = 0.85).

#### 2.3.4 Psychological capital questionnaire (PCQ-12)

The shortened version of the Psychological Capital Questionnaire (PCQ-12) developed by Lorenz et al. (2022) was used. This questionnaire assesses psychological capital as a second-order construct composed of four dimensions: hope (items 1–3), optimism (items 4–6), resilience (items 7–9), and self-efficacy (items 10–12). Items are answered on a six-point Likert scale ranging from “strongly disagree” to “strongly agree,” with higher scores reflecting greater psychological capital.

For use in a Spanish-speaking population, a blind back translation process was carried out following the methodological guidelines of Beaton et al. (2000) and Bracken and Barona (1991), including language validation with the original author and pilot testing in a sample of 15 participants.

The psychometric analyses carried out in the present study indicated high internal consistency for both the total scale (α = 0.93; ω = 0.93) and its dimensions: hope (α = 0.80; ω = 0.80), optimism (α = 0.85; ω = 0.85), resilience (α = 0.79; ω = 0.81), and self-efficacy (α = 0.86; ω = 0.87). Furthermore, the previous study by Lorenz et al. (2022) has confirmed the factorial structure of the scale through confirmatory factor analysis in North American and German samples.

### 2.4 Procedure

This study was approved by the Ethics Committee for Research on Humans of the San Francisco University of Quito (CEISH-USFQ), according to letter No. CE021-2023-CEISH-USFQ and code 2023-021E, dated May 5, 2023. The research was carried out in accordance with the ethical principles established in the 1964 Declaration of Helsinki and its subsequent amendments (World Medical Association, 2013). Participation was completely voluntary and anonymous, and no financial compensation was offered for collaborating in the study.

Data collection took place between October 10, 2023, and January 10, 2024. Participants were recruited via email and mobile messaging invitations after attending a series of webinars organized within the framework of World Mental Health Day. Those who agreed to participate provided informed consent through an online form.

The questionnaires were administered using the ArcGIS web platform, selected for its mobile device compatibility and security measures for data protection. The selection of instruments was based on three key criteria: brevity, psychometric robustness, and accessibility for application in research contexts. The use of this platform minimized the risk of data loss, guaranteed anonymity of responses, and facilitated remote participation, all of which were positively valued by respondents.

### 2.5 Statistical analysis

Statistical analyses were performed using IBM SPSS Statistics, version 26.0 (IBM Corp., Armonk, NY, USA), for descriptive and correlational analyses, and Jamovi, version 2.3.23, for structural equation modeling (SEM) estimation. In the first phase, descriptive statistics of central tendency (mean), dispersion (minimum, maximum, standard deviation), and distribution shape (skewness and kurtosis) were calculated for all variables and dimensions included in the study.

Subsequently, the univariate normality of the variables was examined using the skewness and kurtosis coefficients. Based on these results, Pearson correlations were applied with a significance level of *p* < 0.05 and were interpreted according to Cohen’s (1988) criteria: *r* ≈0.10 (low), *r* ≈0.30 (moderate), and *r* ≥ 0.50 (high).

For the estimation of the structural model, the required statistical assumptions (multivariate normality, homoscedasticity, independence of errors, linearity, and absence of multicollinearity) were previously verified. The model was estimated using the Diagonally Weighted Least Squares (DWLS) method with robust standard errors. The optimization algorithm used was NLMINB, and convergence was achieved in 85 iterations. The Satorra-Bentler corrected fit test was employed for greater estimation precision under non-normal distributions.

To assess model fit, the following indices were considered: the chi-square ratio over degrees of freedom (χ^2^/df), the comparative fit index (CFI), the Tucker-Lewis index (TLI), the root mean square error of approximation (RMSEA), and the standardized root mean square residual (SRMR). The Hu and Bentler (1999) criterion and Byrne (2016) criterion were adopted: χ^2^/df ≤ 3 (acceptable), ≤2 (optimal); CFI and TLI ≥ 0.90 (acceptable), ≥0.95 (excellent); RMSEA and SRMR ≤ 0.08 (acceptable), ≤0.05 (excellent).

## 3 Results

### 3.1 Descriptive

Table 1 presents the descriptive statistics of the variables analyzed, along with their respective dimensions.

**TABLE 1:** Descriptive statistics of the study variables and their dimensions.

Variable	Dimensions	Min	Max	M	M/n	SD	Sk	Ku
Psychological well-being	Self-acceptance	6	36	21.90	3.65	5.55	0.140	−0.68
	Positive relationships	6	36	22.94	3.82	5.54	0.251	−0.138
	Autonomy	13	48	29.26	3.65	4.42	0.376	0.537
	Management of the environment	9	36	23.81	3.96	4.99	0.405	−0.259
	Personal growth	14	42	29.68	4.24	5.70	0.452	−0.731
	Purpose in life	8	36	24.03	4.01	6.03	0.213	−0.467
Spirituality		58	116	92.21	3.18	11.82	0.148	−0.643
	Self-awareness	20	40	32.47	3.25	4.73	−0.182	−0.695
	Spiritual beliefs	8	16	12.32	3.08	2.53	−0.014	−0.934
	Spiritual practices	12	24	17.84	3.57	3.04	0.285	−0.595
	Spiritual needs	18	36	29.58	3.29	3.87	−0.120	−0.470
Psychological capital		13	72	52.60	4.38	11.86	−0.906	0.737
	Hope	3	18	12.68	4.23	3.49	−0.731	0.026
	Optimism	3	18	13.23	4.41	3.29	−0.918	0.732
	Resilience	3	18	12.65	4.22	3.29	−0.662	0.199
	Self-efficacy	3	18	14.04	4.68	3.47	−1.06	0.800

(Min), Minimum; (Max), maximum; (M), mean, (M/n), Mean/number of items; (SD), standard deviation; (Sk), skewness; (Ku), kurtosis.

In relation to psychological well-being, and considering the means prorated according to the number of items in each subscale, it was identified that the dimensions with the highest scores were personal growth and purpose in life, suggesting a favorable perception of individual development and a strong sense of direction with clear objectives, meaning, and substantial goals. However, purpose in life also presented the greatest variability in responses (SD = 6.03), suggesting relevant individual differences in the clarity or stability of this life purpose. In contrast, autonomy showed the least dispersion (SD = 4.42), reflecting greater homogeneity in perceptions related to the capacity for self-determination. Regarding skewness, all dimensions displayed a slight positive skew, denoting a general tendency toward high values with no evidence of extreme bias. Finally, kurtosis was predominantly platykurtic in most dimensions, indicating flatter-than-normal distributions, except for autonomy, whose leptokurtic distribution suggests a greater concentration of scores around the mean.

Regarding spirituality, the results show moderate levels in the total score, indicating that, in general, participants report a significant, although not elevated, connection with transcendent, spiritual, or existential aspects. When analyzing the means prorated by the number of items in each dimension, it was observed that the highest scores were concentrated in spiritual practices and spiritual needs. This finding suggests that individuals tend to actively engage in actions that strengthen their spiritual lives and that they place special importance on the search for inner peace, life purpose, and connection with something greater than themselves. The skewness in the total score and in each of the dimensions was slight, indicating that the response distributions tend to be symmetrical and, therefore, close to normal. Kurtosis was predominantly negative, reflecting platykurtic distributions, that is, flatter than the normal distribution. This characteristic suggests a greater dispersion of responses, which could be interpreted as a diversity of experiences and levels of spirituality among the participants.

Regarding psychological capital, the results show high levels both in the total score and in each of its dimensions: hope, optimism, resilience, and self-efficacy. This profile suggests that, in general, participants perceive they have positive psychological resources that allow them to maintain a confident attitude toward the future, recover from adversity, persist in the achievement of goals, and face challenges with an optimistic attitude. All dimensions of psychological capital presented negative skewness, indicating a concentration of responses at the highest values of the scale. This pattern suggests a general tendency toward high levels of psychological capital in the sample analyzed. On the other hand, the distributions of the total score and its dimensions showed positive kurtosis, implying a greater concentration of responses around the mean, with a lower frequency of extreme values. This statistical behavior suggests relative homogeneity in the perception of these resources among participants, although with some cases with particularly high or low scores, which could represent differentiated individual profiles in terms of psychological strengths.

### 3.2 Bivariate correlations

Table 2 presents the correlations between the dimensions of psychological well-being (self-acceptance, positive relationships, autonomy, environmental management, personal growth, and purpose in life) with the dimensions of spirituality (self-awareness, spiritual beliefs, spiritual practices, and spiritual needs) and psychological capital (hope, optimism, resilience, and self-efficacy). All observed correlations were statistically significant (*p* < 0.001), demonstrating consistent relationships among the variables analyzed in the sample.

**TABLE 2:** Correlations between the study variables and their dimensions.

Variables and their dimensions																
	1	2	3	4	5	6	7	8	9	10	11	12	13	14	15	16
1. Self-acceptance1	1	0.445	0.459	0.722	0.611	0.775	0.470	0.565	0.311	0.317	0.293	0.468	0.470	0.379	0.401	0.386
2. Positive relationships		1	0.254	0.437	0.382	0.340	0.211	0.276	0.123	0.128	0.127	0.171	0.196	0.132	0.141	0.127
3. Autonomy			1	0.408	0.447	0.455	0.259	0.286	0.163	0.178	0.194	0.263	0.242	0.237	0.258	0.184
4. Management of the environment				1	0.654	0.765	0.427	0.487	0.295	0.291	0.288	0.470	0.464	0.406	0.382	0.393
5. Personal growth					1	0.638	0.345	0.361	0.202	0.186	0.334	0.416	0.416	0.344	0.283	0.411
6. Purpose in life						1	0.464	0.512	0.331	0.323	0.321	0.480	0.475	0.394	0.380	0.431
7. Spirituality							1	0.868	0.796	0.829	0.823	0.400	0.370	0.319	0.340	0.368
8. Self-awareness								1	0.622	0.587	0.561	0.406	0.388	0.326	0.367	0.341
9. Spiritual beliefs									1	0.629	0.522	0.267	0.236	0.197	0.246	0.254
10. Spiritual practices										1	0.620	0.269	0.245	0.218	0.252	0.227
11. Spiritual needs											1	0.339	0.311	0.277	0.233	0.363
12. Psychological capital												1	0.875	0.915	0.858	0.857
13. Hope													1	0.748	0.639	0.669
14. Optimism														1	0.761	0.703
15. Resilience															1	0.620
16. Self-efficacy																1

All correlations are significant at the 0.01 level (bilateral).

Regarding the relationship between psychological well-being and spirituality, a strong positive correlation between self-acceptance and spiritual self-awareness stands out, suggesting that people with greater self-acceptance tend to develop a greater connection with their inner self and with transcendent experiences. The dimensions of positive relationships and autonomy showed low, although significant, correlations with spirituality, indicating a less pronounced link. In contrast, the dimensions of environmental management, personal growth, and purpose in life showed moderate to high correlations, mainly with spiritual self-awareness, indicating that individuals with a greater sense of control, personal development, and life orientation also tend to demonstrate greater spiritual awareness and a search for transcendence.

Regarding the relationships between psychological well-being and psychological capital, self-acceptance was moderately associated with all dimensions of psychological capital, particularly with hope and resilience. This suggests that a positive self-image fosters the ability to face adversity with optimism and perseverance. The dimensions of positive relationships and autonomy showed low correlations, indicating a less direct relationship with positive psychological resources. On the other hand, environmental management was moderately related to hope and resilience, implying that the perception of efficacy in managing one’s environment may be linked to the ability to set goals and recover from adverse events. Finally, personal growth and purpose in life showed moderately high correlations with hope and self-efficacy, suggesting that individual development and a focus on meaningful goals are closely related to self-confidence and the motivation to achieve objectives.

Regarding the relationship between spirituality and psychological capital, the results indicate a positive and moderate correlation, suggesting that higher levels of psychological capital are associated with a greater search for meaning, purpose, and transcendent connection. In particular, spiritual self-awareness showed the highest correlations with the four dimensions of psychological capital, highlighting the role that it plays in strengthening resources such as hope, optimism, resilience, and self-efficacy. Spiritual beliefs and practices showed lower, but still statistically significant, associations with the dimensions of psychological capital, while spiritual needs were moderately correlated, suggesting that those with greater psychological resources tend to report greater sensitivity to their spiritual needs, such as inner peace and connection to a life purpose.

### 3.3 Structural model

**FIGURE 1:**
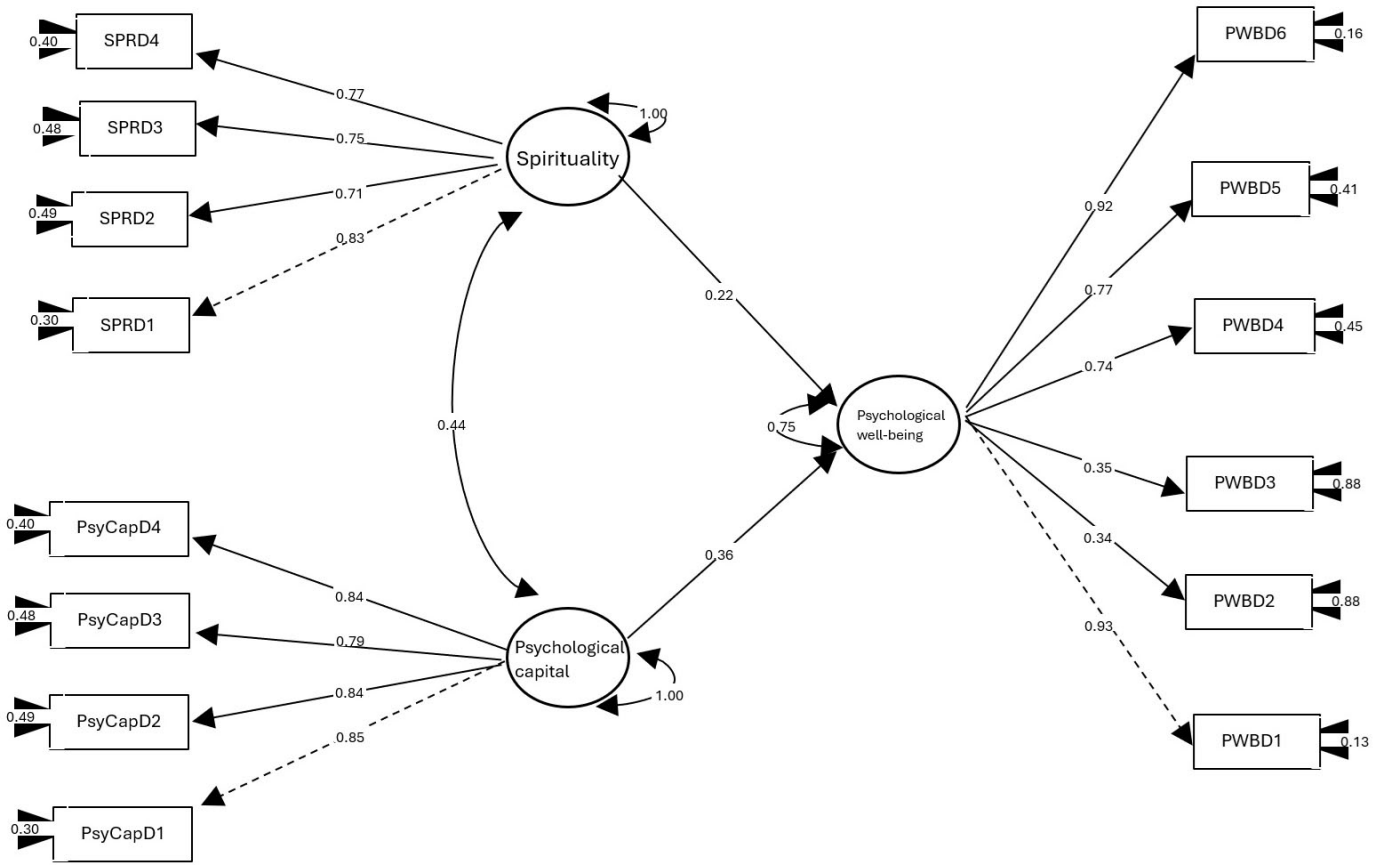
Structural equation model of spirituality, psychological capital and psychological well-being. ESPD1, Self-Awareness; ESPD2, Spiritual Beliefs; ESPD3, Spiritual Practices; ESPD4, Spiritual Needs; PCQD1, Hope; PCQD2, Optimism; PCQD3, Resilience; PCQD4, Self-Efficacy; BPD1, Self-Acceptance; BPD2, Positive Relationships; BPD3, Autonomy; BPD4, Environmental Management; BPD5, Personal Growth; BPD6, Purpose in Life.

The proposed model, represented in Figure 1, showed an adequate fit to the data, according to the following overall indices: CFI = 0.951, TLI = 0.939, GFI = 0.999, NFI = 0.944, RMSEA = 0.079, and SRMR = 0.084. Although the relative chi-square index (CMIN/DF = 7.45, *p* < 0.01) exceeds the suggested optimal values, this result should be interpreted with caution due to its recognized sensitivity to sample size (Hair et al., 2010). However, the combination of the remaining indices supports the overall validity of the model. Furthermore, all estimated beta coefficients were statistically significant (*p* < 0.001), confirming the empirical strength of the proposed relationships.

Figure 1 shows that the psychological well-being indicators presented moderate to high factor loadings. The dimensions of self-acceptance and purpose in life showed the strongest associations with the latent variable, followed by personal growth and environmental management, indicating that these dimensions are especially representative of the construct in this sample. Autonomy and positive relationships also contributed significantly, although with more moderate loadings.

All dimensions of spirituality (self-awareness, spiritual beliefs, spiritual practices and needs) showed high loadings, showing that they constitute reliable and consistent indicators of the spirituality construct.

Similarly, the four dimensions of psychological capital (hope, optimism, resilience, and self-efficacy) performed well as predictors of their latent variable, with statistically significant and theoretically consistent factor loadings. A direct and positive effect of spirituality on psychological well-being was observed, explaining 5% of the latter’s variance. This result suggests that higher levels of spiritual connection, search for meaning, and transcendent practices are associated with psychological well-being.

Psychological capital emerged as the strongest predictor of psychological well-being, explaining 13% of its variance. This relationship reinforces the role of psychological strengths–such as hope, self-efficacy, and resilience–as central factors in promoting well-being from a eudaimonic perspective.

Finally, a positive effect of psychological capital on spirituality was also identified, explaining 19% of its variance. This finding suggests a bidirectional relationship between the two variables, in which personal psychological resources could facilitate connection with transcendent and existential aspects.

The descriptive, correlational, and structural model results empirically support the hypotheses proposed in this study. It is confirmed that both spirituality and psychological capital are positively associated with psychological well-being, with the latter being the most robust predictor. Furthermore, it was shown that psychological capital also significantly influences levels of spirituality, suggesting a dynamic interaction between psychological resources and transcendent experiences. These findings reinforce the theoretical proposition that an individual’s internal strengths play a key role in building a fulfilling and purposeful life, in line with the principles of positive psychology.

## 4 Discussion

The purpose of this study was to analyze the predictive role of spirituality and psychological capital on psychological well-being in a sample of adults from Ecuador and Peru. To our knowledge, no previous research in Latin America (and particularly in these two countries) has examined in a collective manner and through the use of structural equation modeling the direct effect of these two personal strengths on psychological well-being. This contribution is especially relevant in Latin America, given the growing interest in understanding the positive factors that promote human flourishing in diverse sociocultural realities. The findings obtained allowed for the empirical verification of a structural model with adequate fit indices, in which both psychological capital and spirituality significantly predict levels of psychological well-being, thus supporting the proposed hypotheses.

First, the levels of the studied variables were analyzed. Regarding psychological well-being, Ecuadorian and Peruvian participants reported average scores ranging from moderate to high across the six dimensions proposed by Ryff’s (1989), particularly highlighting personal growth and purpose in life. These results reflect the eudaimonic orientation of well-being in this population, characterized by the continuous development of individual potential and the search for meaning in life. This is consistent with the foundations of positive psychology (Ryff, 2023; Jiménez-Lira et al., 2020). However, the observed prorated means are below those reported in previous studies conducted in Spain (Díaz et al., 2006) and Mexico (Medina-Calvillo et al., 2013), although higher than those found in Venezuela (Rondón et al., 2024). These differences could be explained by sociocultural, economic, and contextual factors that influence the experience of well-being. For example, as suggested by Rondón et al. (2024), levels of well-being can fluctuate depending on environmental conditions, including political stability, access to educational opportunities, or social security, which could explain why the scores in this study, despite being high, do not reach the levels recorded in countries with higher levels of human development.

Regarding spirituality, moderate levels were observed, with greater emphasis on the dimensions of spiritual practices and spiritual needs, indicating a spiritual experience focused on the search for inner peace, existential purpose, and transcendent connection, rather than adherence to specific religious beliefs. This orientation is in line with current conceptualizations that understand spirituality as an integrative dimension of the human being, which transcends the religious and acts as a personal resource for coping, meaning, and self-regulation (Koenig et al., 2023; Bożek et al., 2020). The findings are partially consistent with the results of Castañón-Valdivia et al. (2024) and Parra and Vidal-Barrantes (2023), who also observed higher scores in spiritual practices and needs, but less development in the spiritual beliefs dimension. In contrast, the study by Alva and Castillo (2017) reported higher levels of spirituality in medical students, which could be related to vocational factors and training focused on caring for others.

Regarding psychological capital, participants reported high levels of the total score and its four dimensions: hope, optimism, resilience, and self-efficacy. These results are consistent with previous studies conducted in both academic and professional contexts (Siu et al., 2014; Bayona and Guevara, 2019) and support the notion that PsyCap constitutes a key positive resource for facing environmental challenges, fostering adaptation, and promoting well-being (Luthans et al., 2004, 2015; King et al., 2020). Specifically, self-efficacy and optimism were the most highly developed dimensions in this study, suggesting that participants are confident in their ability to achieve goals and maintain a positive attitude toward the future. This psychological strength can be explained, in part, by the profile of the sample (mostly young university students) who, as has been documented in previous research, tend to score higher on the self-efficacy and hope variables (Rodríguez-Cáceres et al., 2018; Saman and Wirawan, 2024).

In the analysis of bivariate correlations, it was observed that all dimensions of psychological well-being presented statistically significant associations with spirituality, and mainly with self-acceptance, environmental management, and purpose in life, which showed stronger relationships with the spiritual self-awareness dimension. These results suggest that people with greater self-acceptance, greater control over their environment, and greater clarity in their goals tend to experience a deeper connection with their inner self, reinforcing the idea that spirituality (conceived as awareness, search for meaning, and transcendence) acts as a positive factor in the construction of psychological well-being (Koenig et al., 2023; Parsian and Dunning, 2009).

These findings are consistent with previous studies in educational and clinical contexts (Bożek et al., 2020; Castañón-Valdivia et al., 2024; Pawar, 2016) that also showed significant relationships between spirituality and psychological well-being, especially in dimensions such as personal growth, purpose in life, and self-acceptance. Specifically, the self-awareness dimension, as a form of introspection and understanding of the self, appears consistently linked to central components of psychological well-being, which has been documented as an important factor in emotional self-regulation, coping, and the perception of life meaning (Joseph et al., 2017; Tanyi, 2002).

On the other hand, the relationship between psychological well-being and capital was consistently evident across all dimensions, although it was particularly notable in the association between self-acceptance and the dimensions of hope and resilience. This reinforces the idea that a positive self-image not only promotes well-being but also enhances the ability to persevere in the face of adversity and maintain a positive attitude toward the future (Bandura, 1997; Luthans et al., 2004). Furthermore, the correlations observed between personal growth and self-efficacy suggest that people oriented toward continuous development tend to be more confident in their ability to achieve goals, which is consistent with previous findings in educational and professional contexts (Siu et al., 2014; Saman and Wirawan, 2024; Jones et al., 2023).

The positive association between spirituality and psychological capital was also statistically significant and of moderate magnitude, especially between spiritual self-awareness and the four dimensions of PsyCap. These results provide further evidence to support the notion that spirituality can act as a psychological resource that strengthens key dimensions of human functioning, such as optimism, hope, resilience, and self-efficacy, by facilitating processes of meaning, emotional regulation, and adaptive coping (Parviniannasab et al., 2022; Caccia and Elgier, 2020). The weaker correlations between PsyCap and spiritual beliefs and practices may indicate that a more reflective and self-connected spiritual experience has a greater impact on the development of these resources than mere doctrinal or ritual adherence.

Finally, the structural equation model empirically supported the proposed hypotheses, demonstrating an adequate fit to the data. Psychological capital (PsyCap) emerged as the strongest predictor of psychological well-being, explaining 13% of its variance, followed by spirituality, which accounted for an additional 5%. These findings align with previous research highlighting the direct influence of PsyCap on well-being in both working populations (Ho and Chan, 2022; Kurt and Demirbolat, 2019) and university students (Saman and Wirawan, 2024). Additionally, a positive effect of PsyCap on spirituality was observed, explaining 19% of its variance, suggesting a potential bidirectional relationship between these constructs and highlighting the capacity of PsyCap to facilitate deeper spiritual development.

While the present results confirm the direct impact of both spirituality and PsyCap on psychological well-being, as well as the influence of PsyCap on spirituality, it is important to consider the possibility of more complex, dynamic relationships. Recent studies support this perspective, proposing that spirituality may act as a mediator or moderator in these associations. For instance, Parviniannasab et al. (2022) and Shamsi Lahijani et al. (2024) demonstrated that spirituality is not only directly linked to mental health and psychological resources but may also contribute to the development of key strengths such as resilience, self-efficacy, and hope, core components of PsyCap.

From this perspective, spirituality, understood as the search for meaning, transcendence, and spiritual self-awareness, may mediate the relationship between PsyCap and psychological well-being by providing an existential framework that enhances the beneficial effects of internal resources on emotional regulation and life purpose. Likewise, it is plausible that spirituality moderates the relationship between PsyCap and well-being, such that the positive impact of psychological resources on well-being is stronger among individuals with higher levels of spiritual development.

Although exploring these mediation and moderation pathways exceeded the scope of the present study, they represent essential avenues for future research. Longitudinal and experimental designs are particularly recommended to advance toward more integrative and dynamic explanatory models of human well-being.

In summary, these results reinforce the postulates of positive psychology regarding the central role of human strengths in building well-being and individual flourishing. Furthermore, the study offers original evidence in the Latin American context, specifically in Ecuador and Peru, where research in this area is still incipient. This contributes to enriching the intercultural understanding of psychological well-being by considering the interaction between spiritual and psychological factors within a contextualized and cross-cultural perspective.

Despite the theoretical and empirical contributions of this study, it is important to consider certain limitations that could have influenced the interpretation of the results. First, the information was collected through self-administered questionnaires, which introduces potential biases related to social desirability, memory errors, and acquiescent responses (Brenner and DeLamater, 2016). This type of measurement, while practical for studies with large samples, can compromise the accuracy of self-reported data.

Second, the cross-sectional design prevents establishing causal relationships between the variables studied. Although structural equation modeling allows for the analysis of complex theoretical relationships, longitudinal or experimental evidence is required to confirm the directionality of the observed effects.

Third, the sample was non-probabilistic and obtained through convenience sampling, which limits the generalizability of the findings. Moreover, there was an imbalance in representation between countries, with a higher proportion of Ecuadorian participants and an overrepresentation of university students. This sampling composition restricts the external validity of the results, particularly when attempting to extrapolate the findings to broader adult populations or other Latin American countries. Nevertheless, the study offers valuable insights into young and emerging adults, a demographic group that is especially relevant for the development of positive psychology interventions targeting psychological well-being, spirituality, and PsyCap.

Based on the limitations identified, several avenues for future research are recommended. First, it is essential to replicate this study using longitudinal designs that allow for examining the directionality, temporal stability, and potential causal relationships among the variables. Second, incorporating mixed-method approaches, including data triangulation with qualitative techniques such as in-depth interviews or focus groups, could provide a more comprehensive understanding of how individuals experience and develop spirituality and psychological capital.

Third, future research should be conducted with more diverse populations, encompassing both urban and rural areas, different age groups, and individuals with varying sociodemographic profiles. Expanding the study to include underrepresented groups, such as older adults, employees in organizational settings, and indigenous communities, would contribute to a broader evaluation of the proposed model’s applicability and cross-cultural validity.

Fourth, it is recommended to explore more complex models, specifically investigating the potential mediating or moderating role of spirituality in the relationship between psychological capital and psychological well-being. Examining these mechanisms could advance our understanding of the dynamic interplay between internal psychological resources and spiritual development.

Finally, it is necessary to design, adapt, and validate intervention programs grounded in positive psychology that simultaneously promote spiritual growth and enhance PsyCap. The effectiveness of such interventions should be rigorously evaluated using experimental or longitudinal methodologies to ensure their practical impact on psychological well-being.

## 5 Conclusion

This study provides novel empirical evidence on the role of spirituality and psychological capital as predictors of psychological well-being in adults from Ecuador and Peru, integrating key constructs from a positive psychology perspective. The results support the hypotheses formulated: first, it was verified that spirituality exerts a direct and positive effect on psychological well-being (H1), reinforcing its role as a source of life meaning, emotional self-regulation, and transcendent connection; second, psychological capital was positioned as the most robust predictor of well-being (H2), highlighting the role of personal resources such as optimism, hope, resilience, and self-efficacy in promoting a fulfilling life; finally, it was also verified that spirituality has a direct effect on psychological capital (H3), suggesting a functional relationship between both variables, where spiritual experience acts as a facilitator for the development of positive psychological strengths.

Altogether, these findings consolidate an integrative explanatory model in which spirituality and psychological capital operate complementarily in the construction of psychological well-being and underscore the importance of fostering these dimensions in young and adult populations from a preventive and mental health-promoting perspective. Furthermore, this study represents an original contribution in the Latin American context, where there is still limited scientific research on these topics, particularly in Andean countries such as Ecuador and Peru. These results open up new lines of research aimed at evaluating psychoeducational interventions that strengthen these internal resources as a way to improve individual and collective well-being in diverse contexts.

## Data Availability

The raw data supporting the conclusions of this article will be made available by the authors, without undue reservation.
